# Prebiotic Inulin and Sodium Butyrate Attenuate Obesity-Induced Intestinal Barrier Dysfunction by Induction of Antimicrobial Peptides

**DOI:** 10.3389/fimmu.2021.678360

**Published:** 2021-06-11

**Authors:** Julia Beisner, Louisa Filipe Rosa, Valentina Kaden-Volynets, Iris Stolzer, Claudia Günther, Stephan C. Bischoff

**Affiliations:** ^1^ Institute of Nutritional Medicine, University of Hohenheim, Stuttgart, Germany; ^2^ Medical Clinic 1, University Hospital Erlangen, Friedrich Alexander University, Erlangen, Germany

**Keywords:** inulin, butyrate, antimicrobial peptides, defensin, innate immunity, obesity, NAFLD

## Abstract

Defects in the mucosal barrier have been associated with metabolic diseases such as obesity and non-alcoholic fatty liver disease (NAFLD). Mice fed a Western-style diet (WSD) develop obesity and are characterized by a diet-induced intestinal barrier dysfunction, bacterial endotoxin translocation and subsequent liver steatosis. To examine whether inulin or sodium butyrate could improve gut barrier dysfunction, C57BL/6 mice were fed a control diet or a WSD ± fructose supplemented with either 10% inulin or 5% sodium butyrate for 12 weeks respectively. Inulin and sodium butyrate attenuated hepatosteatitis in the WSD-induced obesity mouse model by reducing weight gain, liver weight, plasma and hepatic triglyceride level. Furthermore, supplementation with inulin or sodium butyrate induced expression of Paneth cell α-defensins and matrix metalloproteinase-7 (MMP7), which was impaired by the WSD and particularly the fructose-added WSD. Effects on antimicrobial peptide function in the ileum were accompanied by induction of β-defensin-1 and tight junction genes in the colon resulting in improved intestinal permeability and endotoxemia. Organoid culture of small intestinal crypts revealed that the short chain fatty acids (SCFA) butyrate, propionate and acetate, fermentation products of inulin, induce Paneth cell α-defensin expression *in vitro*, and that histone deacetylation and STAT3 might play a role in butyrate-mediated induction of α-defensins. In summary, inulin and sodium butyrate attenuate diet-induced barrier dysfunction and induce expression of Paneth cell antimicrobials. The administration of prebiotic fiber or sodium butyrate could be an interesting therapeutic approach to improve diet-induced obesity.

## Introduction

An increased intake of energy-dense foods that are high in fat and added sugars, a so called Western-style diet (WSD), is strongly associated with obesity and related metabolic diseases (1). A high-fat and high-sugar diet contributes to the development of insulin insensitivity and dyslipidemia (2–4). Insulin resistance and dyslipidemia lead to an increase in hepatic lipid accumulation which further contributes to the development of non-alcoholic fatty liver disease (NAFLD) and type 2 diabetes (5). Due to the increasing incidence of obesity, which represents an important risk factor for NAFLD and diabetes, the prevalence of NAFLD is rising and is emerging as one of the most common cause of chronic liver disease (6).

Animal and human studies revealed that a WSD influences the intestinal barrier function and this impairment is associated with the development of obesity and metabolic syndrome (7, 8). A WSD can cause epithelial barrier dysfunction and increased intestinal permeability, which facilitates the translocation of bacteria and bacterial components such as the endotoxin lipopolysaccharide (LPS) to the blood stream leading to metabolic endotoxemia (9–11). Antimicrobial peptides (AMPs) play a key role in intestinal barrier function by protecting the host against pathogen infections and translocation of commensal bacterial antigens, and by regulating the healthy microbiota composition. While α-defensins are mainly expressed and stored in secretory granules of Paneth cells in the small intestine (12), β-defensins are produced by intestinal epithelial cells including the colonic mucosa (13). Paneth cells secrete a variety of AMPs, among these high levels of α-defensins HD-5 and HD-6 (14). α-defensins in mice, termed cryptdins, are the major microbicidal components of mouse Paneth cell granules. Among these, cryptdin-4 is the most potent bactericidal peptide *in vitro* (15). To obtain antimicrobial activity, proteolytic activation of murine α-defensin precursors by matrix metalloproteinase-7 (MMP7), which colocalizes in Paneth cell granules, is required (16, 17). Mouse Paneth cells also uniquely express cryptdin-related sequences (CRS)-peptides, an additional related family of antimicrobial peptides (18).

Recent evidence reporting a decreased α-defensin HD-5 protein expression in the jejunum of obese subjects compared with normal weight controls has provided a link between altered Paneth cell function and obesity (19). Besides preventing bacterial translocation in the healthy intestine, Paneth cells act as a second line of defense by limiting bacterial translocation in intestinal barrier loss. A compromised antimicrobial Paneth cell defense has been thus associated with increased bacterial translocation (20–22). We previously demonstrated that fructose-supplemented Western-style diet not only leads to an increased gut permeability, endotoxemia and subsequent hepatic steatosis in mice but hampers expression of Paneth cell-specific α-defensin-1 suggesting that Paneth cells might play a role in diet-induced translocation of bacteria (8). Administration of human recombinant HD-5 improves metabolic dysfunction in diet-induced obese mice by reversing dyslipidemia and alleviating markers of liver damage and jejunal inflammation pointing towards a possible therapeutic potential of defensins in improving NAFLD (23).

While high-sugar and high-fat consumption is associated with a disturbed intestinal barrier function, the intake of prebiotics and sodium butyrate is thought to improve intestinal integrity and the antimicrobial peptide defense (24–26) offering an new strategy to treat obesity and related metabolic disorders. In various mouse models, the administration of inulin, which is fermented into short chain fatty acids (SCFA) such as butyrate, propionate or acetate, was able to reduce pro-inflammatory processes, improve intestinal integrity and promote the intestinal microbiota composition and diversity (27–29) but mechanisms supporting these associations remain largely unknown. Therefore, in this study, we investigated the effects of inulin and sodium butyrate on antimicrobial defense and intestinal barrier function in a mouse model of diet-induced obesity and examined whether inulin or sodium butyrate could improve gut barrier dysfunction and liver steatosis.

## Material and Methods

### Experimental Setup, Animals, and Diets

Female C57BL/6 mice were housed in a specific pathogen-free (SPF) barrier facility with a fully controlled environment and 12-hour light/dark cycles accredited by the Association for Assessment and Accreditation for Laboratory Animal Care International. Experiments were carried out with female C57BL/6 mice as they have been shown to be more susceptible to fructose-induced nonalcoholic fatty liver disease (30). Mice aged 6-8 weeks were fed *ad libitum* a control diet (CD, Ssniff E15000-04), a western-style diet (WSD, ssniff, TD88137-modified) or a WSD supplemented with inulin at 10% weight/weight (26, 27) (ssniff, S0514-E780) or sodium butyrate at 5% weight/weight (26, 31) (ssniff, S0514-E782) (**Table 1**) *ad libitum* for 12 weeks. Autoclaved tap water either with or without supplementation of D- (–)-fructose 30% weight/weight (F, >99.5% purity, Carl Roth, Karlsruhe, Germany) was offered ad libitum to the mice (8).

**Table 1:** Primers used in quantitative real-time PCR.

	Forward (5’-3’)	Reverse (5’-3’)
*Actb*	GCTGAGAGGGAAATCGTGCGTG	CCAGGGAGGAAGAGGATGCGG
*Defa1*	TCAAGAGGCTGCAAAGGAAGAGAAC	TGGTCTCCATGTTCAGCGACAGC
*Defa21*	CCAGGGGAAGATGACCAGGCT	TGCAGCGACGATTTCTACAAAGGC
*Defa29*	CACCACCCAAGCTCCAAATACACAG	ATCGTGAGGACCAAAAGCAAATGG
*pan-cryptdin*	AAGAGACTAAAACTGAGGAGCAGC	GGTGATCATCAGACCCCAGCATCAGT
*Mmp7*	TTCAAGAGGGTTAGTTGGGGGACTG	CCGCCTCTACGAGTGAAACTGTT
*Lyz1*	GCCAAGGTCTACAATCGTTGTGAGTTG	CAGTCAGCCAGCTTGACACCACG
*Nod2*	GGCACCTGAAGTTGACATTTTGC	ATCTCCCACAGAGTTGTAATCC
*Reg3g*	TTCCTGTCCTCCATGATCAAAA	CATCCACCTCTGTTGGGTTCA
*Myd88*	CAAAAGTGGGGTGCCTTTGC	AAATCCACAGTGCCCCCAGA
*Defb1*	TCCAATAACATGCATGACCA	TCATGGAGGAGCAAATTCTG
*Ocln*	ACTCCTCCAATGGACAAGTG	CCCCACCTGTCGTGTAGTCT
*Cldn2*	GTCATCGCCCATCAGAAGAT	ACTGTTGGACAGGGAACCAG
*Cldn5*	GCTCTCAGAGTCCGTTGACC	CTGCCCTTTCAGGTTAGCAG
*ZO-1*	CCACCTCTGTCCAGCTCTTC	CACCGGAGTGATGGTTTTCT
*Muc2*	GATGGCACCTACCTCGTTGT	GTCCTGGCACTTGTTGGAAT
*Tnf*	ACCACCATCAAGGACTCA	AGGTCTGAAGGTAGGAAG
*Il1b*	ACGGATTCCATGGTGAAGTC	GAGTGTGGATCCCAAGCAAT
*Il6*	AGTCACAGAAGGAGTGGCTA	CTGACCACAGTGAGGAATGT
*Gpr41/Ffar3*	GAAAAGAGTGGACCGCAGGA	AGGGCAATCTGAGTGACAGC
*Gpr43/Ffar2*	TTCTTACTGGGCTCCCTGCC	TACCAGCGGAAGTTGGATGC
*Gpr109/Hcar*	CTGCTCAGGCAGGATCATCTGGAG	CCCTCTTGATCTTGGCATGT

Food intake, fluid intake, and body weight were assessed weekly. After 12 weeks, barrier tests were performed. Then, animals were anesthetized with ketamine-xylazine (100:16 mg/kg body wt) by intraperitoneal injection. Blood was collected by portal vein puncture just before the mice were killed. Liver and gut tissue specimens were collected and frozen immediately in liquid nitrogen as well as preserved in neutral-buffered formalin or Carnoy’s solution for histomorphological investigation. Experiments were approved by the local Animal Care and Use Committee (Regional Council Stuttgart 312/14 EM).

### RNA Isolation and Quantitative Real-Time PCR

Total RNA was isolated from tissue using the peqGOLD TriFast system (PEQLAB, Erlangen, Germany). A total of 500 ng RNA was reverse transcribed using the Reverse Transcription System kit and random primers after a DNase digestion step (Promega, Madison, WI). To determine the gene expression RT-PCR was performed using EvaGreen^®^ Supermix SsoFastTM (Bio-Rad Laboratories GmbH, München, Germany) in a 10 µl reaction mixture containing cDNA, SYBR Green Master Mix and mouse-specific oligonucleotides primers (**Table 1**). β-Actin was used as a reference gene. The amplification reaction was carried out in an iCycler (Bio-Rad Laboratories) with an initial hold step (95°C for 3 min) and 45 cycles of a three-step PCR (95°C for 15 s, 60°C for 15 s, and 72°C for 30 s) followed by a post-PCR dissociation curve performed between 60°C and 95°C. Transcript copy numbers of cryptdin-1, cryptdin-4, CRS1C, MMP7, lysozyme, Reg3γ and mBD-1 were determined by comparison with a quantitative standard curve generated by serial dilution of plasmid standards and normalized to the transcript copy numbers of mouse β-actin. The relative gene expression of pan-cryptdin, IL-6, IL-1β, ZO-1, occludin, mucin 2, claudin-2, claudin-5, Myd88, GPR41,GPR43 and GPR109a was calculated using the ΔΔ-Ct method in comparison to the house keeping gene β-actin.

### Intestinal Permeability and Urinary Claudin-3

After the 12-week diet phase mice were fasted for 6h before the intestinal permeability test using polyethylene glycol 4000 (PEG4000) was carried out. The precise procedure including dosing, sample collection and detection of markers by HPLC were recently described by Volynets et al. (32). Concentrations of claudin-3 in urine were determined using a high-sensitive ELISA assays (SEF293Mu; Hölzl) as described previously (32). Colorimetric analyses were carried out using a spectrophotometer (λ = 500 nm and 37°C) (Multi-Detection Microplate Reader, Synergy TM HT; Bio-Tek) and Gen5 version 2.01.

### Endotoxin/Lipopolysaccharide (LPS) Measurement

For determination of endotoxin, portal vein blood was collected, heparinized, and diluted 1:50. Endotoxin concentrations were determined with the use of a limulus amebocyte lysate kinetic assay (LAL Endosafe Endochrome-K, R1710K; Charles River) as previously described (32).

### TG in Plasma and Liver Tissue

Plasma TGs were determined with the use of a commercial kit and lipid control level 2 (TR 210 and LE 2662; Randox Laboratories). Colorimetric analyses were carried out using a spectrophotometer (λ = 500 nm and 37°C) (Multi-Detection Microplate Reader, Synergy TM HT; Bio-Tek) and Gen5 version 2.01. For determination of hepatic triglyceride concentrations liver tissue samples (50–100 mg) were homogenised in ice-cold double-concentrated PBS. Tissue lipids were extracted with methanol (100ml)–chloroform (200 ml), dried and resuspended in 5% fat-free bovine serum albumin. Colorimetric assessment of TG concentrations was carried out using the Randox Laboratories Kit. Values were normalized to protein concentrations determined using the Bradford assay in the liver homogenates.

### HE Staining and Immunofluorescence Staining

Paraffin sections of liver and gut tissue (5 µm) were stained with hematoxylin/eosin for histological evaluation as previously described (33). For fluorescence-microscopy, 3 µm thick paraffin sections of the ileum were prepared. Paraffin-embedded samples were deparaffinized and rehydrated. For immunofluorescence analysis sections were blocked with 5% normal donkey serum for 1h and incubated with DEFA5 primary antibody (1:100; PAB912Mu01, dianova GmbH, Hamburg) overnight at 4°C. Donkey Anti-Rabbit Alexa Fluor 594 (145043, Jackson ImmunoResearch Laboratories, Baltimore) was used as secondary antibody (diluted 1:800). Tissue sections were counterstained with 4′,6-diamidino-2-phenylindole (DAPI) (1:10000 diluted in PBS) to visualize nuclei. After DAPI staining, sections were mounted with Fluoromount-G (Southern Biotechnology Associates, Birmingham, USA) for microscopy examination. The sections were analyzed using Axiovert 200M ZEISS fluorescence microscope (Carl Zeiss Microscopy, Oberkochen, Germany). Defa5 immunofluorescence was quantified by optical density measurements using AxioVision 4.8.2 SP3 software. The total area of the Defa5 fluorescence signal (F2) was determined in a fixed frame. The determined area (in pixels) was then related as a percentage (F2/F4) to the total area of the defined frame (F4). For each slide, 5 images were created, evaluated as described, and the mean value was calculated.

### Localization of Bacteria by Fluorescent *In Situ* Hybridization (FISH)

To analyze bacteria localization at the surface of the intestinal mucosa, intestinal mucin and adjacent bacteria were visualized with the use of mucin 2 (Muc2) immunofluorescence (primary antibody: H-300 sc-15334; Santa Cruz Biotechnology) combined with fluorescent *in situ* hybridization with the use of the 16S rRNA EUB338 probe (5#-GCTGCCTCCCGTAGGAGT-3# with a cyanine 3 label; biomers.net GmbH). Ileal sections were treated with a hybridization solution (20 mM Tris-HCl; 0.9 M NaCl; 0.1% SDS; 30% formamide) and incubated overnight at 50°C. The sections were washed in FISH wash buffer (20 mM TrisHCl; 0.9 mM NaCl) and blocked with 5% BSA in PBS. Afterwards samples were incubated with the first antibody (Mucin 2 (H-300) sc-15334; Santa Cruz Biotechnology, Dallas, USA) and a second blocking solution (1% BSA in PBS) at 4°C overnight. Alexa Fluor^®^ 488 (goat anti-rabbit IgG (H+L) Life technologies, Carlsbad, USA) was used as secondary antibody and nuclei were stained with DAPI (Carl Roth GmbH & Co KG, Karlsruhe, Germany). The slides were mounted with the use of an antifade mounting medium (Vectashield H-1000; Vector Laboratories). Tissue sections were analyzed with the use of an Axiovert 200M ZEISS fluorescence microscope (Carl Zeiss) at a magnification of 200 times.

### Organoid Culture

Small intestinal organoids were isolated from the mouse small intestine and cultured with ENR medium (organoid medium with epidermal growth factor/Noggin/R-spondin) for a minimum of seven days according Sato et al. (34). Organoid growth was monitored by light microscopy. Organoids were stimulated with sodium butyrate, sodium acetate, sodium D-lactate and sodium propionate (Sigma-Aldrich, Germany) for 30 h. To investigate the HDAC inhibitory effect of butyrate, organoids were stimulated with MS275 or HDAC-8-IN (35).Total RNA was extracted from organoids using the peqGOLD Microspin Total RNA Kit (Peqlab, Erlangen, Germany) and complementary DNA (cDNA) was synthesized using the SCRIPT cDNA Synthesis Kit (Jena Bioscience, Jena, Germany).

### Statistical Analysis

The statistical analysis was performed using GraphPad Prism software 7.0 (GraphPad Software Inc., La Jolla, USA). For the statistical comparison of more than two groups a one-way analysis of variance, Kruskal-Wallis-test for non-parametric data followed by Dunn’s test or one-way-ANOVA and Sidak´s post-test was performed. Differences between two groups were analyzed by using unpaired t-test or Mann-Whitney test as appropriate. P-values of p < 0.05 were considered as statistically significant.

## Results

### Inulin and Sodium Butyrate Attenuate Weight Gain and Hepatic Steatosis in WSD-Fed Mice

**Figure 1:**
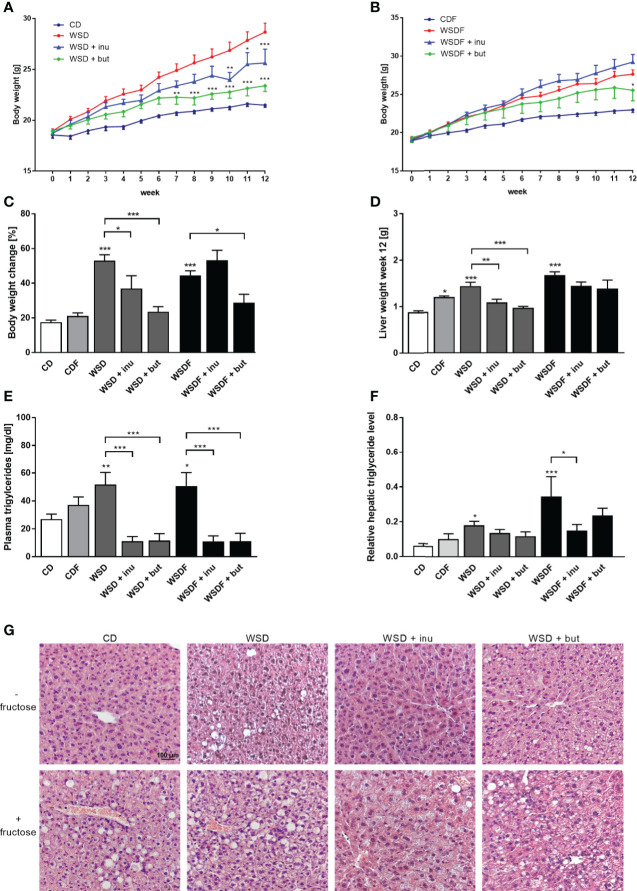
Inulin and sodium butyrate reduce body weight and liver weight gain and attenuate hepatic steatosis in WDF-fed mice. C57BL/6 mice fed either a control diet (CD), a Western-style diet (WSD) or a WSD supplemented with 10% inulin (inu) or 5% sodium butyrate (but) with or without additional fructose **(F)** are shown. Weight development of mice fed diets without fructose **(A)** and with fructose **(B)** during the intervention of 12 weeks. Body weight change **(C)**, liver weight **(D)**, triglyceride concentration in plasma **(E)** and hepatic triglyceride level relative to protein concentration in liver tissue **(F)** after the 12-week intervention period. **(G)** Representative images of liver specimens stained with hematoxylin and eosin. Scale bar: 100 µm. Data are presented as means +/- standard error of the mean (SEM) (n = 6–8). Statistical analysis was performed by 2-way ANOVA with Tukey’s multiple comparisons test **(A, B)** or one-way ANOVA with Sidak´s post-test. Significant differences are indicated as *p-value < 0.05; **p-value < 0.01; ***p-value < 0.001.

Inulin and sodium butyrate significantly attenuated body weight gain of WSD-fed mice whereas in mice receiving a WSDF for 12 weeks, only sodium butyrate significantly reduced body weight (**Figures 1A–C**). This effect was not caused by a reduced energy intake nor differences in water intake, since inulin and sodium butyrate did not change the calorie intake and the water intake over 12 weeks (**Figure S1**). Mice fed a WSD or WSDF showed higher plasma triglyceride level which were significantly reduced when inulin or sodium butyrate were supplemented (**Figure 1E**).

We next evaluated the effect of inulin and sodium butyrate treatment on hepatic steatosis markers such as liver weight, hepatic lipid accumulation and hepatic triglyceride levels. Inulin and sodium butyrate supplementation decreased the liver weight gain in mice fed a WSD but not in mice fed a WSDF (**Figure 1D**). WSD-fed mice, and in particular fructose-rich diet-fed mice, exhibited a pronounced increase in hepatic lipid accumulation which was reduced by inulin and sodium butyrate supplementation (**Figure 1G**). Moreover, inulin- and sodium butyrate-treated mice exhibited improvements in liver lipid metabolism substantiated by lower levels of hepatic triglycerides in WSDF-fed mice (**Figure 1F**).

### WSD Induces a Decrease in Paneth Cell Antimicrobials Which Is Reversed by Inulin and Sodium Butyrate

**Figure 2:**
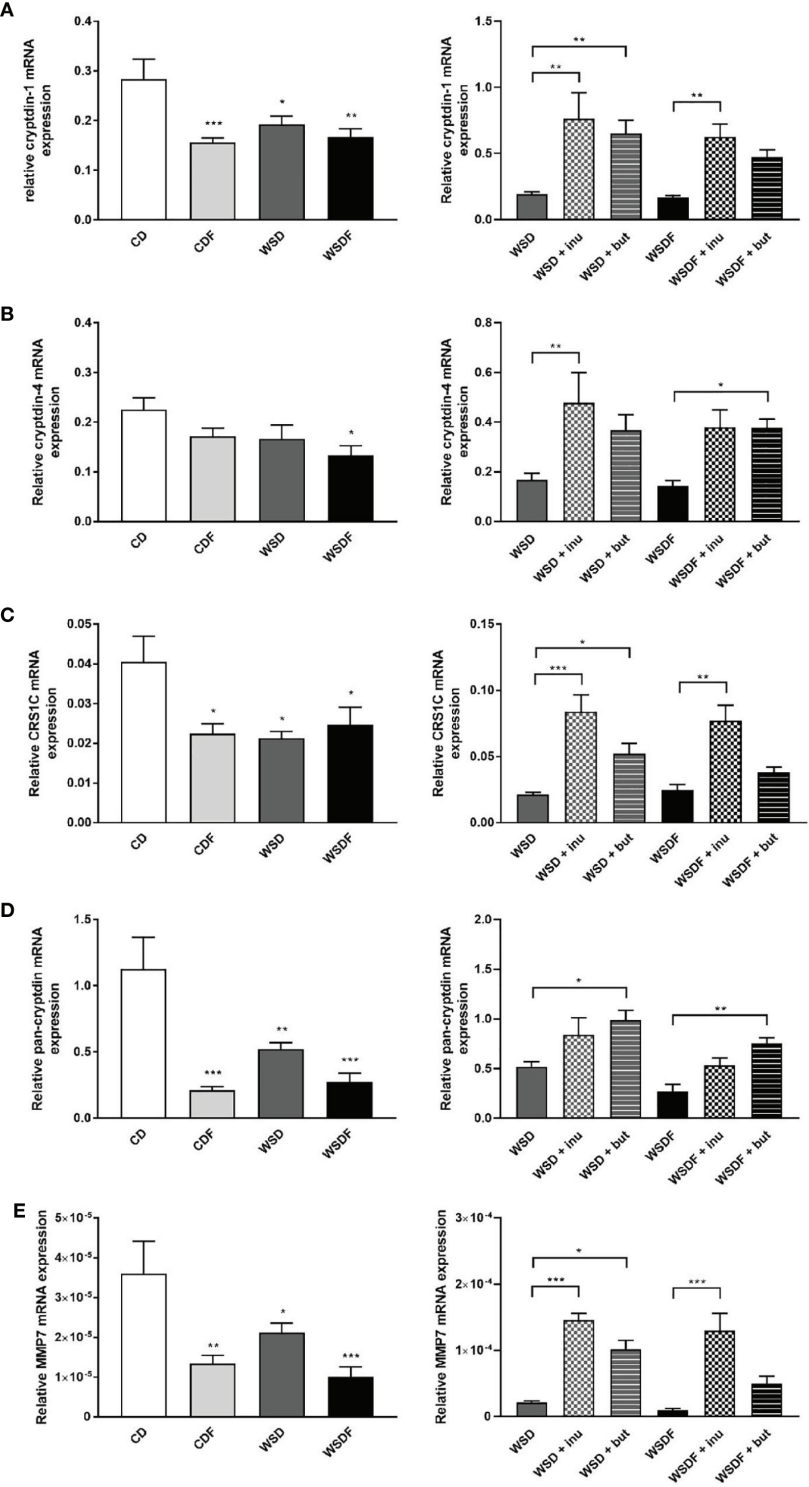
Inulin and sodium butyrate induce Paneth cell antimicrobial expression which is decreased by high-fat and high-sugar diet. C57BL/6 mice fed different diets as described in **Figure 1** are shown. Relative mRNA expression level of cryptdin-1 **(A)**, cryptdin-4 **(B)**, CRS1C **(C)**, pan-cryptdin **(D)** and matrix metalloproteinase-7 (MMP7) **(E)** in ileal tissue determined by quantitative RT-PCR. Data are presented as means +/- standard error of the mean (SEM) (n = 6–8). Statistical analysis was performed by one-way ANOVA with Dunnett´s or Sidak´s post-test. Significant differences are indicated as *p-value < 0.05; **p-value < 0.01; ***p-value < 0.001.

To examine the role of Paneth cell antimicrobials in diet-induced obesity we determined mRNA expression of different α-defensins. WSD, either with or without additional fructose feeding, caused a significant decrease of α-defensin gene expression in the ileum. Cryptdin-1 (Defa1) mRNA expression and CRS1C mRNA expression were significantly decreased in mice fed a CDF, WSD or WSDF (**Figures 2A–C**). Cryptdin-4 (Defa21) mRNA expression level were significantly reduced in the WSDF group (**Figure 2B**). Total Paneth cell cryptdin expression was strongly diminished in mice fed a WSD, and particularly in mice challenged with additional fructose (CDF, WSDF) (**Figure 2D**). No difference in Paneth cell number was observed in mice fed a high-fat and high-sugar diet indicating that the decreased expression of antimicrobial proteins was not caused by reduced cell numbers (data not shown). Supplementation of the WSD and WSDF with sodium butyrate and in particular with inulin led to an induction of Paneth cell α-cryptdin-1, cryptdin-4 and CRS1C mRNA expression, which was mostly statistically significant (**Figures 2A–C**). Ileal expression of total Paneth cell cryptdins was significantly induced in mice who received a sodium butyrate supplemented WSD or WSDF whereas inulin was less effective (**Figure 2D**). Together these data support an important role of ileal α-defensins in mediating the effects of sodium butyrate and inulin on antimicrobial barrier function.

Next, we aimed at determining whether alterations in Paneth cell α-defensins activation might be associated with the protective effect of inulin and sodium butyrate. MMP7 proteolytically converts pro-α-defensins into their mature and active forms and thus plays an important role in defensin activity (16). Ileal MMP7 mRNA expression was significantly decreased in the CDF, WSD and WSDF-fed mice as compared to CD-fed mice (**Figure 2E**) suggesting a critical role of MMP7 in the diet-induced impairment of the antimicrobial defense. In mice fed a WSD without additional fructose supplementation, MMP7 expression was less affected. Following administration of inulin and sodium butyrate we observed a strong induction of ileal MMP7 mRNA expression (**Figure 2E**) suggesting that inulin and butyrate might be involved in the activation of α-defensins.

**Figure 3:**
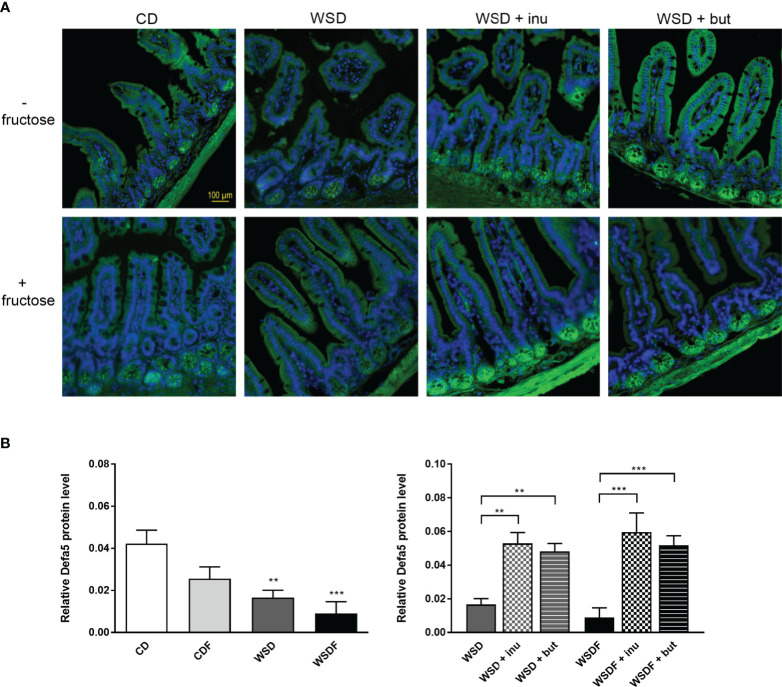
Supplementation with inulin or sodium butyrate induces Defa5 protein expression in ileal Paneth cells. C57BL/6 mice fed different diets as described in **Figure 1** are shown. **(A)** Representative images of Defa5 immunofluorescence staining in ileal tissue showing Defa5 expression in green and cell nuclei detected by DAPI in blue. Scale bar: 100 µm. **(B)** Quantification of Defa5 expression. Data are presented as means +/- standard error of the mean (SEM). Statistical analysis was performed by one-way ANOVA with Dunnett´s or Sidak´s post-test. Significant differences are indicated as **p-value < 0.01; ***p-value < 0.001.

Within Paneth cells, antimicrobial peptides are stored in cytoplasmatic granules, from which they can be released into the gut lumen, thereby contributing to intestinal host defense. Immunofluorescence analysis revealed Defa5 protein expression in Paneth cell granules of mice fed a control diet whereas Defa5 staining was substantially reduced in the ileal crypts of mice fed a WSD and almost completely depleted in WSDF fed mice (**Figure 3A**). We also noted a downregulation of α-defensin 5 in the ileal crypts of mice fed a fructose-supplemented CD though this effect was not statistically significant in the quantitative analysis (**Figure 3B**). Supplementation of the WSD and WSDF with inulin or sodium butyrate induced Defa5 protein expression in ileal Paneth cells. Sections stained with anti-Defa5 antibody showed strong immunoreactivity of Paneth cells. Quantitative analysis revealed that both, inulin and sodium butyrate, significantly increased the expression of Defa5 in Paneth cells of mice fed a supplemented WSD and WSDF compared to mice fed a WSD and WSDF without supplementation (**Figure 3B**). These results indicate that inulin or sodium butyrate restore ileal Defa5 expression *in vivo*

### Supplementation With Inulin and Sodium Butyrate Induces an Increase of Other Antimicrobial Peptides and Colonic β-Defensin-1

**Figure 4:**
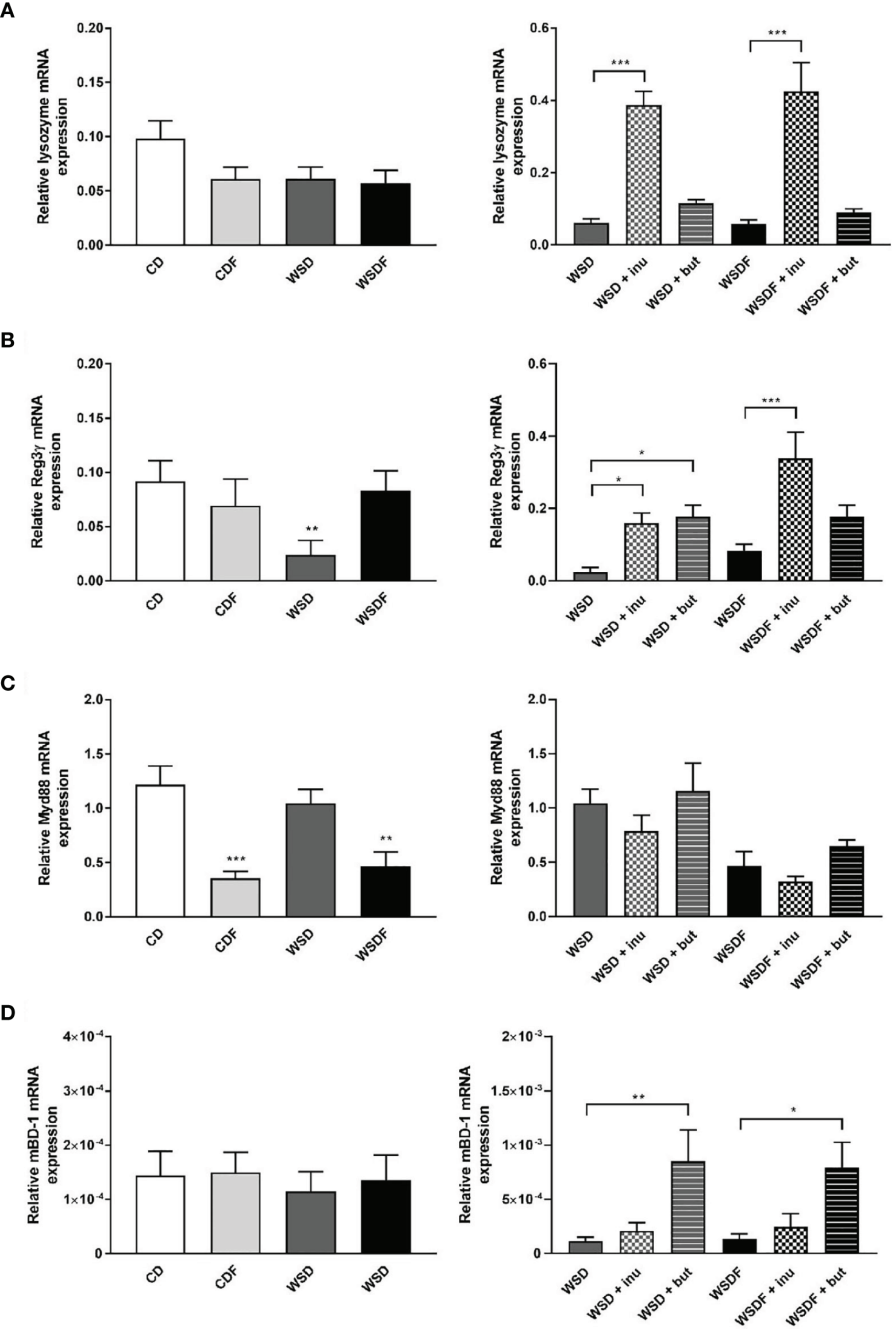
Effects of inulin and sodium butyrate on antimicrobial peptide expression. C57BL/6 mice fed different diets as described in **Figure 1** are shown. Relative mRNA expression level of lysozyme **(A),** Reg3γ **(B)**, MyD88 **(C)** in the ileum and mBD-1 **(D)** in the colon determined by quantitative RT-PCR. Data are presented as means +/- standard error of the mean (SEM) (n= 6–8). Statistical analysis was performed by one-way ANOVA with Dunnett´s or Sidak´s post-test or by Kruskal-Wallis-test for non-parametric data with a Dunn’s post-test. Significant differences are indicated as *p-value < 0.05; **p-value < 0.01; ***p-value < 0.001.

Paneth cells also produce several other antimicrobial peptides (36). We further analyzed expression of lysozyme and Reg3γ, which are abundantly expressed in Paneth cells. In contrast to α-defensin expression, we observed no significant effect of diets on lysozyme mRNA expression (**Figure 4A**). However, supplementation with the prebiotic fiber inulin led to a strong induction of lysozyme whereas sodium butyrate had no effect on its expression level (**Figure 4A**). Reg3γ was specifically reduced in WSD-fed mice which could be reversed by both inulin and sodium butyrate supplementation (**Figure 4B**). The data indicate that Paneth cell antimicrobials are differentially regulated.

Paneth cells directly sense enteric bacteria through MyD88-dependent toll-like receptor (TLR) activation, triggering expression of multiple antimicrobial factors (20). Interestingly, MyD88 mRNA expression was significantly reduced in mice fed fructose-rich diets, CDF and WSDF but not in mice fed a WSD (**Figure 4C**). Supplementation with inulin and sodium butyrate though did not significantly affect MyD88 mRNA level (**Figure 4C**). In contrast, nucleotide-binding and oligomerisation domain (NOD) 2, which might regulate Paneth cell function, was up-regulated by supplementation of inulin and sodium butyrate to the high-fat and high-sugar diet (**Figure S2**).

In the colon, β-defensins secreted by the colonic epithelium represent the major innate host defenses. The constitutive expression of β-defensin-1 prevents microbial invasion by strengthening the intestinal mucosal barrier (37). Therefore, we examined gene expression of mouse β-defensin-1 in the colonic mucosa. Colonic mBD-1 mRNA expression was in general of low levels and unaffected by high-fat and high-sugar diets (**Figure 4D**). Nevertheless, supplementation with sodium butyrate led to a strong induction of mBD-1 mRNA expression in the colonic mucosa (**Figure 4D**).

### Inulin and Sodium Butyrate Reduce Luminal Bacteria in the Ileum

**Figure 5:**
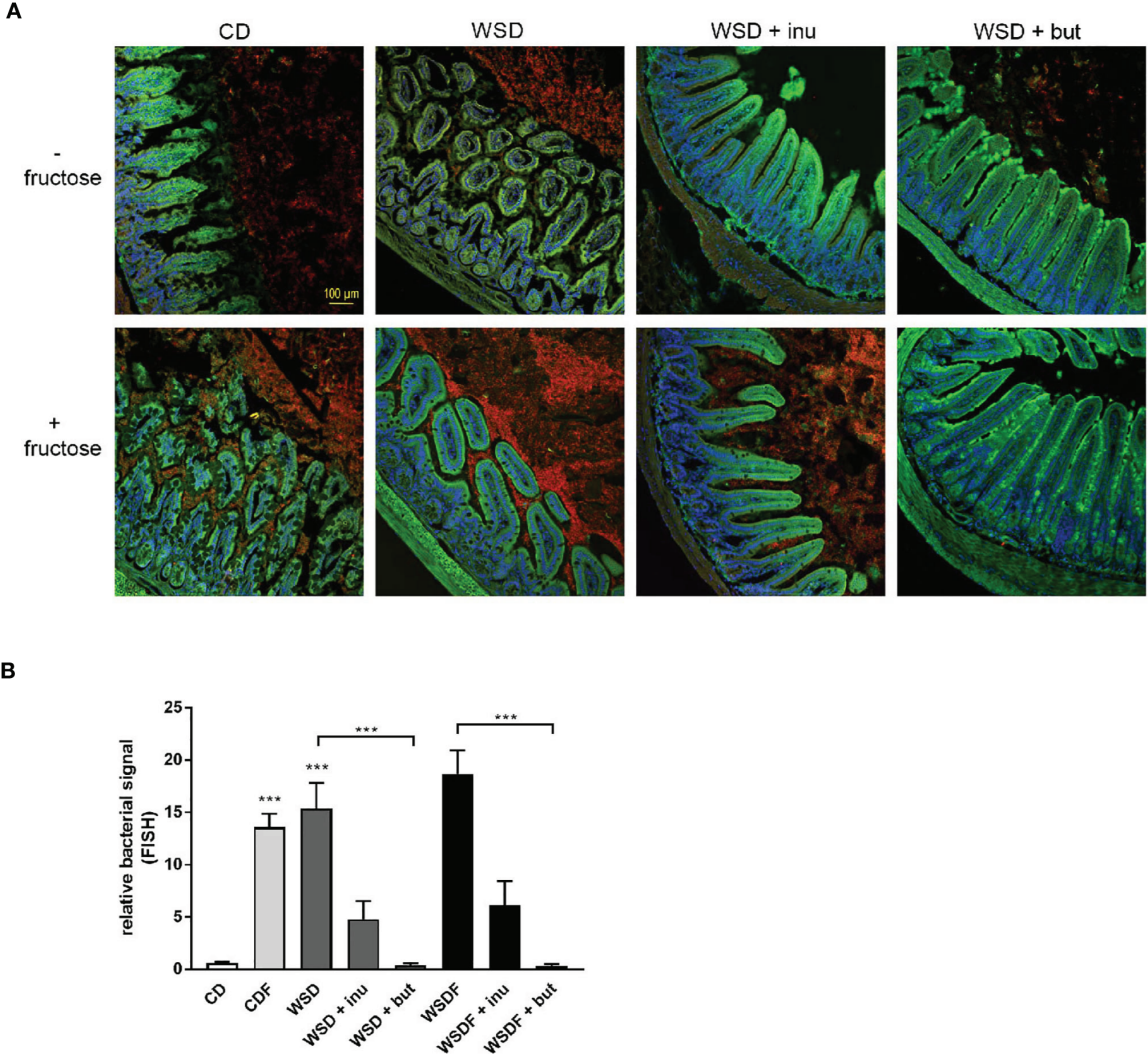
Effects of inulin and sodium butyrate on small intestinal bacterial overgrowth (SIBO). C57BL/6 mice fed different diets as described in **Figure 1** are shown. **(A)** Representative images of FISH staining in ileal tissue showing bacterial growth in red and cell nuclei detected by DAPI in blue. Scale bar: 100 µm. **(B)** Quantification of bacterial overgrowth. Data are presented as means +/- standard error of the mean (SEM). Statistical analysis was performed by one-way ANOVA with Kruskal-Wallis-test for non-parametric data with a Dunn’s post-test. Significant differences are indicated as ***p-value < 0.001.

In mice fed a control diet, mucin staining revealed that the area between the villi was largely filled with mucus. In addition, the villus area was free of bacteria, as shown by quantification of bacterial rRNA (**Figure 5A**). In contrast, feeding a WSD and a high fructose consumption resulted in a bacterial overgrowth. Particularly with increased fructose consumption (CDF and WSDF), the number of bacteria between the villi also increased strongly (**Figure 5A**). The supplementation with sodium butyrate or inulin resulted in a lower occurrence of bacteria between the villi compared to the WSD- and WSDF-fed mice without sodium butyrate or inulin (**Figure 5A**). Quantitative analysis revealed that sodium butyrate significantly reduced the bacterial signal in mice fed a supplemented WSD and WSDF (**Figure 5B**). Supplementation with inulin also reduced the bacterial signal in the ileum of WSD and WSDF-fed mice though effects were not significantly different.

### Effect of Inulin and Sodium Butyrate Supplementation on Intestinal Tight Junctions

**Figure 6:**
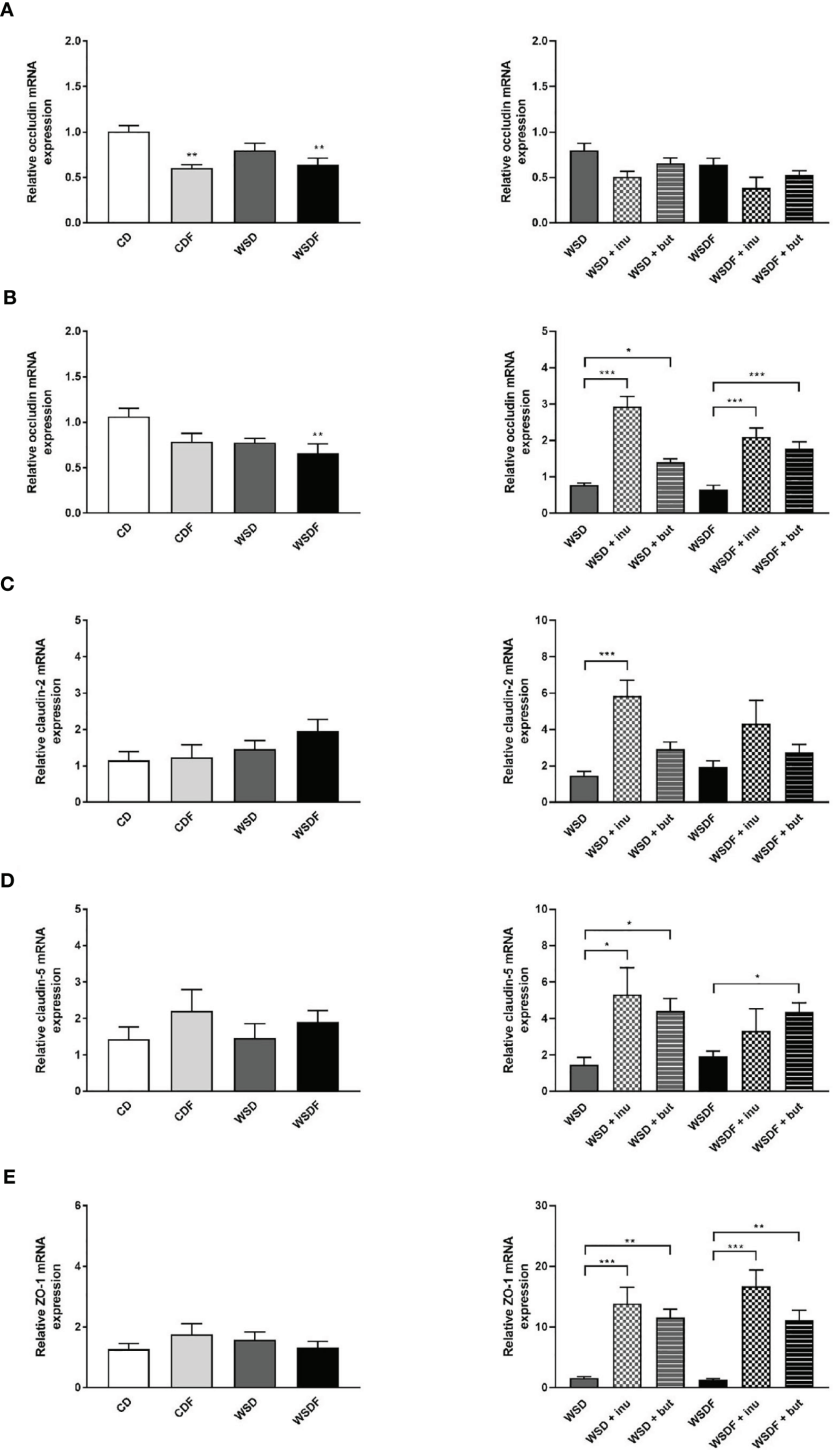
Effect of inulin and sodium butyrate supplementation on intestinal tight junctions. C57BL/6 mice fed different diets as described in **Figure 1** are shown. Relative mRNA expression level of occludin in the ileum **(A)** and in the colon **(B)**, claudin-2 **(C),** claudin-5 **(D)** and ZO-1 **(E)** in the colon determined by quantitative RT-PCR. Data are presented as means +/- standard error of the mean (SEM) (n = 6–8). Statistical analysis was performed by one-way ANOVA with Dunnett´s or Sidak´s post-test or by Kruskal-Wallis-test for non-parametric data with a Dunn’s post-test. Significant differences are indicated as *p-value < 0.05; **p-value < 0.01; ***p-value < 0.001.

Since tight junctions (TJ) maintain the enteral epithelial barrier and their defects may result in gut impairment, we analyzed the impact of high-fat and high-sugar diet and supplementation with inulin and sodium butyrate on the components of TJ expressed in the ileum and colon. As reported earlier, occludin mRNA expression levels were significantly lower in the ileum of mice fed fructose-rich diets CDF and WSDF and in the colon of mice which received a WSDF (**Figures 6A, B**). Inulin and sodium butyrate did not affect occludin expression in the ileum but strongly increased expression of occludin in the colon in mice which received the inulin and butyrate supplemented WSD or WSDF (**Figure 6B**). In mice fed a WSD an increased claudin-2 mRNA expression was observed following inulin supplementation (**Figure 6C**). Moreover, claudin-5 mRNA expression was significantly increased in the WSD + inulin and WSD + sodium butyrate group compared to the WSD diet group as well as in the WSDF + sodium butyrate group compared to the WSDF group (**Figure 6D**). Similarly, zonula occludens protein-1 (ZO-1) mRNA expression was significantly induced by inulin and sodium butyrate supplementation in WSD or WSDF fed mice (**Figure 6E**). Mucin 2 mRNA abundance was not influenced neither by high-fat and high-sugar diet nor by supplementation with inulin or sodium butyrate (**Figure S3**). Altogether these results suggest that inulin and sodium butyrate may contribute to the restoration of the colonic tight junction barrier by abrogating the high-fat and high sugar-induced downregulation of occludin.

### Supplementation With Inulin and Sodium Butyrate Improves Intestinal Permeability

**Figure 7:**
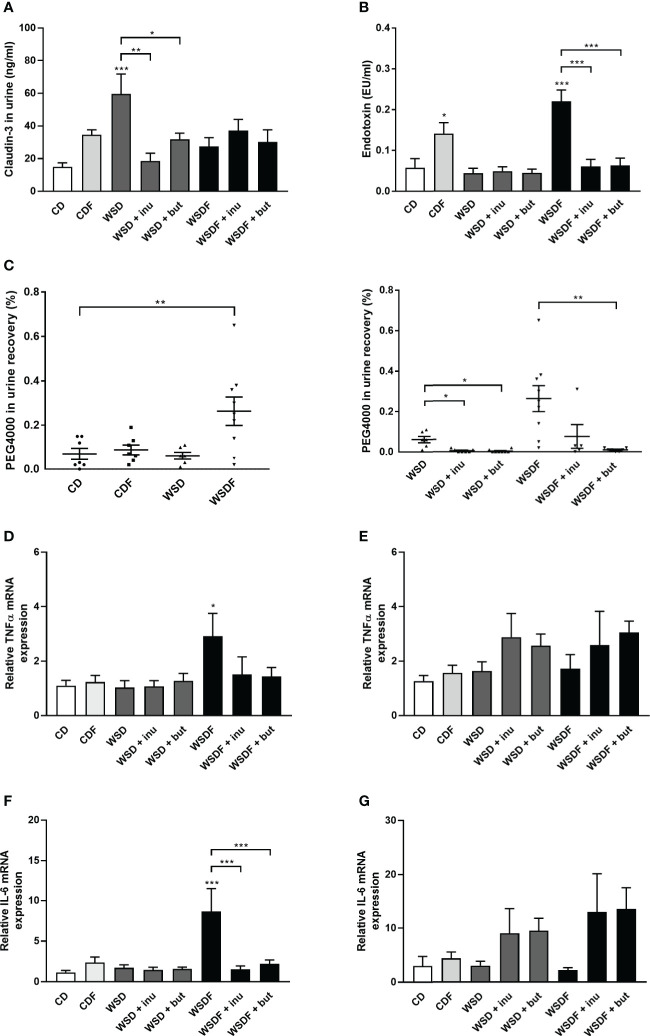
Supplementation with inulin and sodium butyrate improves intestinal permeability and inflammation. C57BL/6 mice fed different diets as described in **Figure 1**. **(A)** Intestinal permeability was determined by claudin-3 level in the urine. **(B)** Endotoxin concentrations in the portal venous plasma. **(C)** PEG4000 recovery in urine in percent of orally administered PEG4000 by gavage. Relative mRNA expression level of TNF**-**α **(D)** and IL-6 **(F)** in the ileum and TNF-α **(E)** and IL-6 **(G)** in the colon determined by quantitative RT-PCR. Data are presented as means +/- standard error of the mean (SEM) (n = 6–10). Statistical analysis was performed by one-way ANOVA with Dunnett´s or Sidak´s post-test or by Kruskal-Wallis-test for non-parametric data with a Dunn’s post-test. Significant differences are indicated as *p-value < 0.05; **p-value < 0.01; ***p-value < 0.001. PEG4000, polyethylene glycol 4000.

The above described results prompted us to examine claudin-3 level in the urine, a non-invasive marker for intestinal tight junction loss and intestinal permeability (38). WSD caused elevated concentrations of claudin-3 in the urine which were significantly reduced by inulin and sodium butyrate supplementation (**Figure 7A**). We next assessed intestinal permeability using the high-MW polyethylene glycol 4000 (PEG4000) which does not cross the rodent mucosal barrier without pronounced intestinal barrier impairment. The analysis revealed an increased permeability in mice that had received a WSDF (**Figure 7C**). WSD-fed mice supplemented with inulin or sodium butyrate showed a significant decrease in intestinal absorption of the PEG marker compared to WSD-fed mice (**Figure 7C**). Furthermore, the strongly increased permeability induced by the WSDF was abolished by supplementation with inulin or sodium butyrate (**Figure 7C**) suggesting that inulin and sodium butyrate strengthen the intestinal epithelial barrier.

Previous work has shown that high-fat and high-sugar diet increases plasma endotoxin (8, 11). We therefore analyzed whether high-fat and high-sugar diets promote bacterial translocation and endotoxemia and whether inulin or butyrate supplementation could alleviate endotoxemia. Mice fed the fructose-rich diets, CDF and WSDF, showed significantly elevated plasma endotoxin levels (**Figure 7B**). Supplementation with inulin and butyrate significantly attenuated the plasma endotoxin levels, exerted by feeding a WSDF.

Pro-inflammatory cytokine tumor necrosis factor α (TNF-α) and IL-6 mRNA expression was significantly increased in the ileum of the WSDF-fed mice which was normalized by inulin or sodium butyrate supplementation (**Figures 7D–F**). In the colon TNF-α level and IL-6 mRNA level tended to be generally higher but were not significantly changed by inulin or sodium butyrate supplementation (**Figures 7E–G**). IL-1β- mRNA expression was not different in the ileum but elevated by inulin and butyrate supplementation in colon of WSD-fed mice (**Figure S4**).

### Inulin and Sodium Butyrate Counteract GPR43 Overexpression Induced by WSD

One pathway by which short-chain fatty acids (SCFA) may exert their effects is through the activation of orphan G-protein–coupled receptors. Short-chain fatty acids (SCFA) can activate G-protein-coupled receptors 41 (GPR41), 43 (GPR43) and 109a (GPR109a) (39, 40) which are expressed in mouse intestinal epithelial cells and Paneth cells (41, 42). Inulin-derived SCFA as well as sodium butyrate may thus function through the activation of GPR41, GPR43 and GPR109a receptors and regulate expression of Paneth antimicrobials *via* this mechanism. We therefore determined expression of GPR41, GPR43 and GR109a receptors. GPR43 mRNA abundance in the ileum was significantly up-regulated in CDF, WSD and WSDF-fed mice compared with mice fed a control diet (**Figure S5B**), whereas no significant effect was observed on GPR41 mRNA expression (**Figure S5A**). Dietary supplementation of inulin or sodium butyrate to the WSDF significantly decreased the GPR43 mRNA expression in the ileum (**Figure S5B**). There was no significant difference in GPR43 level between mice fed a WSD or a WSD supplemented with inulin or sodium butyrate which might be attributed to the originally lower levels of GPR43 in WSD-fed mice. In the colon, inulin and sodium butyrate led to an increase in GPR41 (**Figure S5C**) and GPR109 (**Figure S5E**). The mRNA expression of GPR109a in the ileum was not detectable. These data demonstrate that the effects of SCFAs on Paneth cell antimicrobials might not be directly mediated by an activation of GPR41/GPR43 in the ileum, whereas GPR41 and GPR109a might play a role in mediating protective effects of SCFAs in the colon.

### SCFAs Induce Paneth Cell A-Defensin Expression in Organoids *In Vitro*

**Figure 8:**
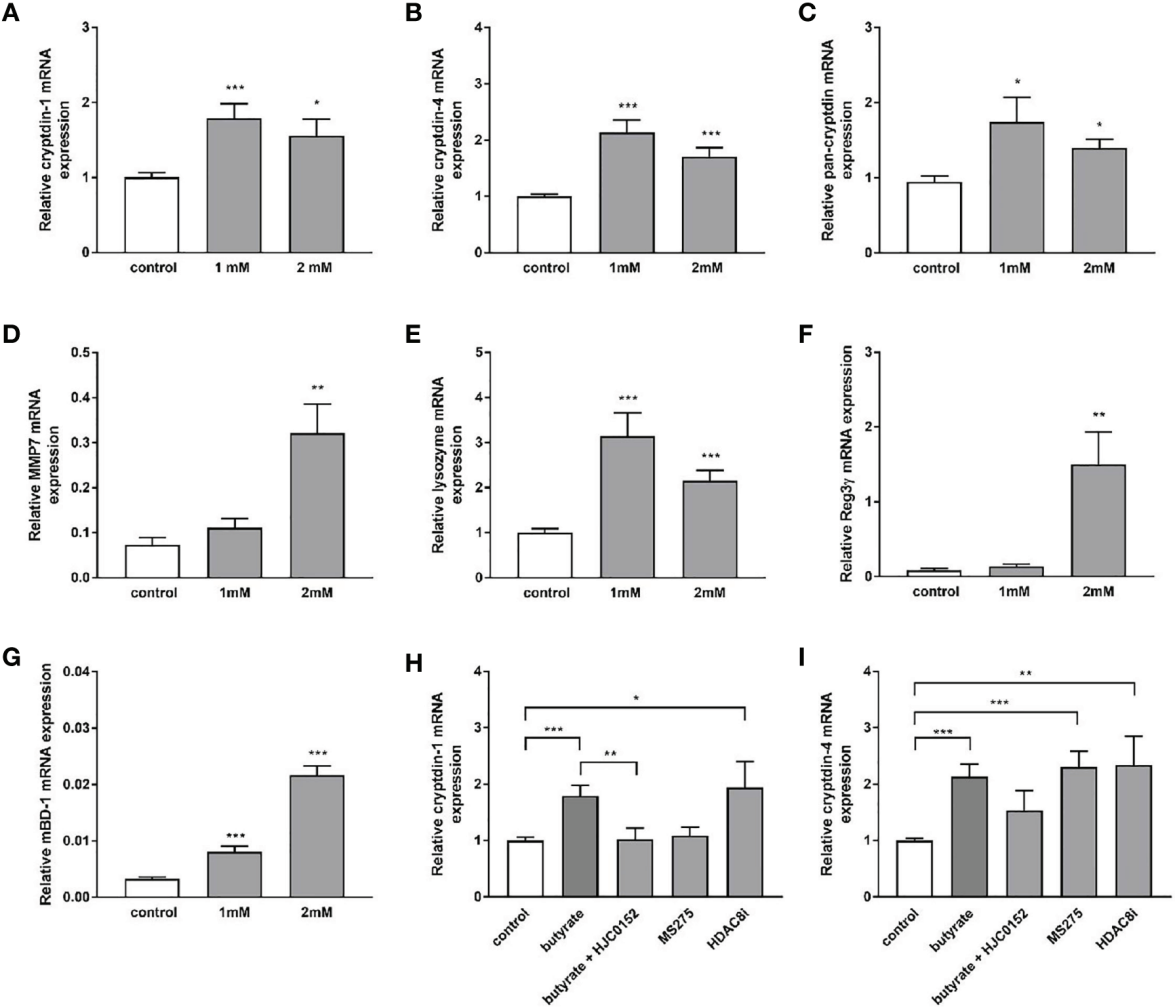
Sodium butyrate induces expression of antimicrobial peptides in murine organoids *in vitro*. Organoids were treated with 1mM and 2mM sodium butyrate for 30h. Relative mRNA expression level of cryptdin-1 **(A),** cryptdin-4 **(B),** pan-cryptdin **(C)**, MMP7 **(D)**, lysozyme **(E)** Reg3γ **(F)**, mBD-1 **(G)** determined by quantitative RT-PCR derived in organoids from the small intestine of healthy C57BL/6 mice (n=6). Organoids were treated with sodium butyrate (1mM), sodium butyrate in combination with HJC0152 (5μM) or MS-275 (2μM) or HDAC8-IN-1 (0.5μM). Relative mRNA expression level of cryptdin-1 **(H)** and cryptdin-4 **(I)** determined by quantitative RT-PCR derived in organoids from the small intestine of healthy C57BL/6 mice (n=3-6). Data are presented as means ± SEM and were analyzed by unpaired t-test **(A–G)** or one-way ANOVA with Sidak´s post-test **(H, I)**. Significant differences are indicated as *p-value < 0.05; **p-value < 0.01; ***p-value < 0.001.

**Figure 9:**
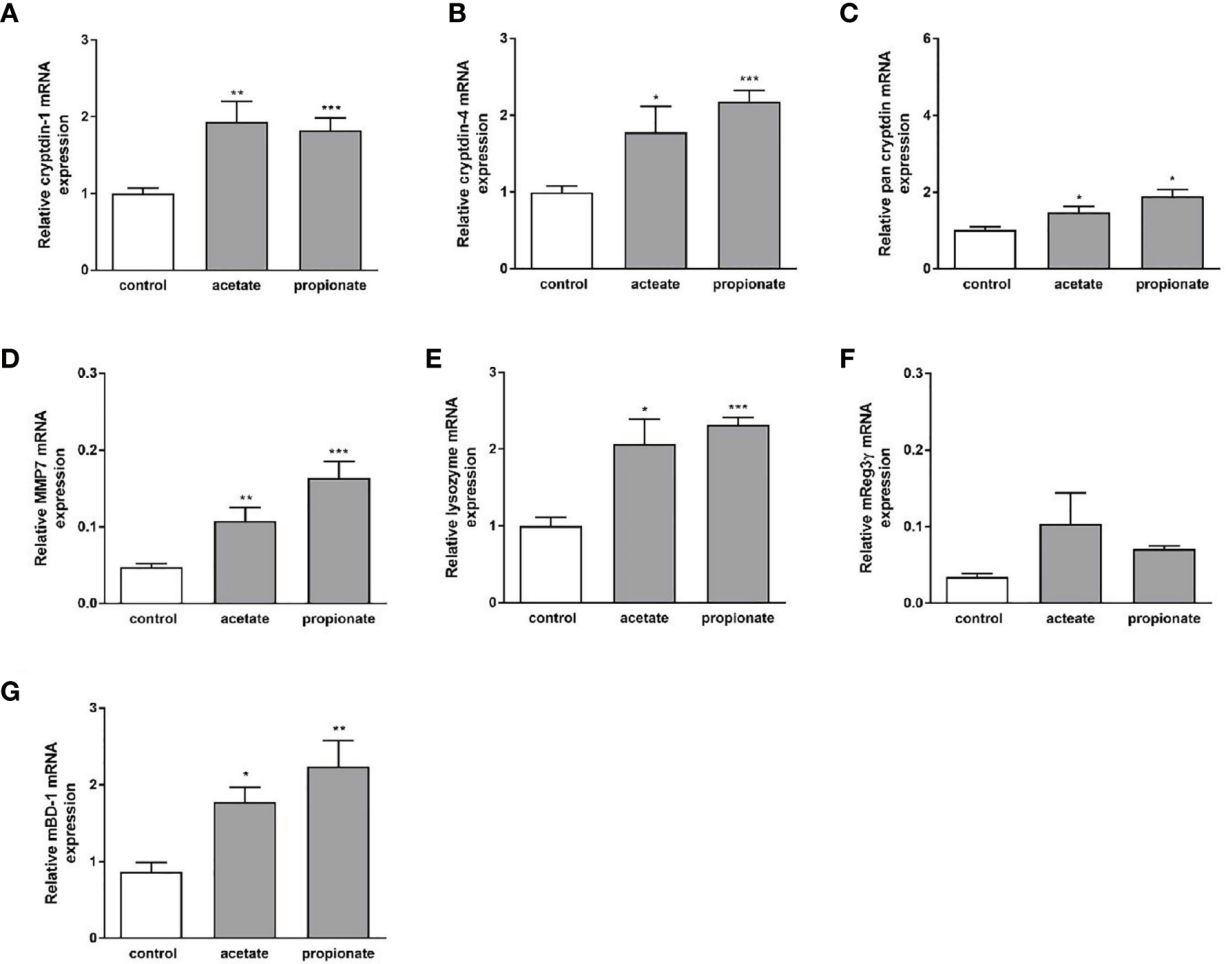
Effect of sodium propionate and acetate on expression of antimicrobial peptides in murine organoids *in vitro*. Organoids were treated with sodium propionate (1mM) or sodium acetate (1mM) for 30h. Relative mRNA expression level of cryptdin-1 **(A),** cryptdin-4 **(B)**, pan-cryptdin **(C)**, MMP7 **(D)**, lysozyme **(E)** Reg3γ **(F)**, mBD-1 **(G)** determined by quantitative RT-PCR derived in organoids from the small intestine of healthy C57BL/6 mice (n=3-6). Data are presented as means ± SEM and were analyzed by unpaired t-test. Significant differences are indicated as *p-value < 0.05; **p-value < 0.01; ***p-value < 0.001.

Dietary fibers such as inulin are fermented by gut bacteria into short-chain fatty acids, mainly acetate, propionate, and butyrate (43). Effects of inulin on Paneth cell host defense may therefore be partly mediated by short chain fatty acids (SCFAs) produced through fermentation of inulin by the intestinal microbiota. To explore whether SCFA can directly regulate Paneth cell cryptdin expression *in vitro*, we carried out organoid cultures of small intestinal crypts as previously described (44). Intestinal organoids from healthy C57BL/6 mice were treated with sodium butyrate, sodium propionate and sodium acetate for 24 h. Administration of sodium butyrate resulted in a significant induction of cryptdin-1 (**Figure 8A**), cryptdin-4 (**Figure 8C**) and pan-cryptdin mRNA expression. Similarly, the administration of sodium propionate and acetate resulted in an induction of α-defensin mRNA expression (**Figures 9A–C**). Interestingly, MMP7 mRNA expression level were also significantly augmented by sodium butyrate (**Figure 8D**), propionate and acetate (**Figure 9D**) suggesting that short chain fatty acids not only affect expression of Paneth cell α-defensin but also the conversion of the inactive pro-α-defensin precursors into their active form. The Paneth cell antimicrobial lysozyme was also induced by sodium butyrate (**Figure 8E**), propionate and acetate (**Figure 9E**). In line with previous results we observed that mBD-1 and Reg3γ mRNA expression was strongly increased by sodium butyrate (**Figures 8F, G**), whereas sodium propionate and acetate only induced mBD-1 (**Figure 9G**) and not Reg3γ mRNA expression (**Figure 9F**). These data suggest that short chain fatty acids could be causally involved in the protective effect of inulin.

Butyrate is a well-known histone deacetylase inhibitor and selectively inhibits class I HDAC and IIa activity (45). We hypothesized that the inhibition of HDACs could promote the expression of Paneth cell antimicrobials. To verify this hypothesis, organoids were treated *in vitro* with the HDAC inhibitor MS-275 and HDAC8-IN-1. Treatment of intestinal organoid cultures with MS-275 and HDAC8-IN-1 induced cryptdin-4 expression to a comparably strong effect like sodium butyrate (**Figure 8I**). In contrast cryptdin-1 expression was not changed by MS-275 treatment but significantly increased by inhibition of HDAC8 (**Figure 8H**). These findings suggest an important role of histone deacetylation in the transcriptional regulation and inducibility of α-defensins. Butyrate treatment of mouse small intestinal epithelial cells leads to activation of STAT3 (46). We hypothesized that STAT3 might regulate SCFA induction of α-defensin expression. The specific STAT3 inhibitor HJC015233 significantly attenuated butyrate-induced cryptdin-1 mRNA expression (**Figure 8H**) and also reduced cryptdin-4 expression though to a lower extent suggesting that STAT3 is critical for the butyrate-induced cryptdin expression.

## Discussion

Alterations in the intestinal barrier function resulting in an increased translocation of bacterial products contribute to the development of NAFLD and other metabolic diseases. The prebiotic fiber inulin is a possible candidate to ameliorate metabolic disorders exerting beneficial effects through alleviating endotoxemia and inflammation. Inulin and its fermentation product butyrate have been shown to promote mucosal barrier integrity and function in mice and humans. However, the molecular mechanisms underlying the regulation of the intestinal barrier are not fully understood. In the present study, we provide evidence that inulin and sodium butyrate supplemented to a WSD attenuate intestinal barrier dysfunction by induction of Paneth cell α-defensins. Our study reveals for the first time that inulin and sodium butyrate strengthen Paneth cell antimicrobial function in diet-induced obese mice *in vivo* and highlights the potential role of HDAC inhibition and STAT3 signaling in mediating the effect of inulin and its fermentation products on α-defensins.

Dietary supplementation of inulin and sodium butyrate significantly inhibited the body weight gain induced by WSD. Impressively, inulin and sodium butyrate supplementation prevented elevated plasma triglyceride level and plasma LPS level despite the fact that mice were fed a WSD or WSDF during a 12-week intervention period. The attenuation of body weight and hepatic lipid accumulation is a likely reason for the improved lipid profile induced by inulin and sodium butyrate. Our results are in line with previous findings which reported that dietary inulin significantly reduced body weight, serum triglyceride and plasma LPS in inulin-treated mice (27, 47). It is possible that apart from reinforcement of the intestinal barrier function, changes in microbiota composition may also contribute to the observed lower portal plasma LPS levels in inulin-supplemented mice. Reduced translocation of LPS might prevent LPS-induced infiltration of inflammatory cells in the intestinal ileum as corroborated by reduced ileal expression of pro-inflammatory TNF-α. This is consistent with our previous results showing that inulin supplementation attenuated TNF-α level in the ileum of Casp8^ΔIEC^ knockout mice (26).

In the present study, we provide evidence that the protective effect of inulin and sodium butyrate supplementation on the intestinal barrier is mediated at least partially through an enhanced expression of tight junction molecules. Similarly, Chen et al. found higher levels of the TJ protein occludin in inulin-type fructan-fed mice (29). Similar beneficial effects have been reported for the short chain fatty acid butyrate by inducing tight junction assembly of ZO-1 and occludin (48, 49). Occludin depletion leads to a preferential increase in the flux of large macromolecules (50) which substantiates our observations of a reduced intestinal permeability of PEG4000 in mice fed an inulin or sodium butyrate supplemented high-fat and high-sugar diet. In contrast to our results, the study by Zhang et al. (51). found no effects of an inulin-supplemented diet on the expression of tight junction genes in the ileum which might be attributed to a lower inulin content in the diet and a shorter dietary treatment compared to our protocol. The findings that inulin strengthens the intestinal mucosal barrier integrity were extended in a recent study showing that supplementation with inulin at low concentration prevented increased mucus penetrability in WSD-fed mice (52).

Our data indicate that inulin and sodium butyrate supplementation induced ileal expression of Paneth cell cryptdins, cryptdin-4 and -1 and CRS-peptide in mice fed a WSD supporting an important role of α-defensins in mediating the effects of sodium butyrate and inulin on antimicrobial barrier function. Our results are in line with previous findings from a sclerosis mouse model showing that HD-5 and lysozyme mRNA expression were recovered by administration of butyric acid in the drinking water (53). We found that the Paneth cell antimicrobial lysozyme was induced by inulin but not affected by sodium butyrate supplementation hinting at a different regulation that might be influenced by host–gut microbiota interactions. The fact that inulin and sodium butyrate also induced MMP7 expression suggests that the mechanism of α-defensin activation is likely involved in restoring the antimicrobial barrier function. The improved α-defensin expression combined with higher MMP7 level in the ileum points towards a causal role of defensin function in mediating the protection of gut immunity in inulin and butyrate supplemented diet-induced obese mice. Moreover, alteration in α-defensin function by inulin and sodium butyrate might represent an additional potential mechanism in regulating gut microbiota composition. Increased antimicrobial peptide expression in the ileum might also impact the gut barrier function in the colon. Studies revealed that mouse Paneth cell α-defensins secreted into the small intestinal lumen persist in an intact and functional form in the colonic lumen suggesting that the peptides may mediate enteric innate immunity in the colon, far from their upstream point of secretion in small intestinal crypts (54). Although expression of α-defensin has been shown to be up-regulated by commensal bacteria in the small intestine in a MyD88-dependent manner (55), inulin and sodium butyrate did not significantly affect MyD88 expression in diet-induced obese mice.

Paneth cells act as a second line of defense by restricting the contact between resident luminal microbes and mucosal surfaces and limiting the number of LPS bearing bacteria and LPS molecules coming into close contact with the apical side of intestinal epithelial cells (13). Paneth cell antimicrobials have microbicidal activity against luminal bacteria, including gram-negative bacteria, such as Escherichia coli, Salmonella typhimurium (56). We have shown in previous studies that intestinal bacterial translocation in liver cirrhosis is related to compromised Paneth cell antimicrobial host defense in the small intestine (22) suggesting that a decrease in Paneth cell products results in reduced antimicrobial activity in the distal ileum and impacts bacterial translocation. Furthermore, Salzman et al. have demonstrated that Paneth cell defensins can inhibit bacterial translocation in a transgenic mouse model (57). In a rat model of endotoxemia bacterial translocation was most prominent in the ileum and was correlated with mucosal inflammatory response to LPS (58). We therefore strongly propose that increased Paneth cell antimicrobial peptide production in the small intestine induced by inulin and sodium butyrate reduces the number of LPS bearing bacteria and LPS molecules coming into contact with the mucosa and thus inhibits endotoxin translocation.

In butyrate-supplemented diet-induced obese mice up-regulation of α-defensin expression in the small intestine was paralleled by the induction of β-defensin-1 expression in the colon, which is critical in the maintenance of epithelial barrier function (37). Our results are in line with previous findings that short-term butyrate feeding after antibiotic treatment promotes expression of β-defensin 1 in a GPR43-dependent manner (46). Human β-defensin-1-derived net formations of its reduced form have been shown to inhibit bacterial translocation (59) indicating that by this additional mechanism of action it provides a further barrier for commensals and pathogens.

Inulin and sodium butyrate exerted similar effects on expression of Paneth cell antimicrobials, other antimicrobial peptides and tight junctions as well as on plasma triglycerides, independent of whether they were supplemented to a WSD or a fructose-rich WSD. WSDF-fed mice were characterized by a specific decrease of Paneth cell antimicrobials and tight junctions indicating that fructose supplementation has an additive effect. Notably, body weight and liver weight gain were attenuated by inulin or sodium butyrate in WSD-fed mice, whereas in mice fed a WSD with additional fructose liver weight gain was not significantly reduced. The rise in portal vein endotoxin levels in mice fed a fructose-rich WSD, a major trigger for liver steatosis (8), was attenuated by inulin and sodium butyrate supplementation suggesting that inulin and sodium butyrate might have favorable effects not only on hepatic lipid accumulation but also on bacterial translocation induced by the high-fructose diet. Although the particular harmful effects of adding fructose to the usual western, high-fat and high-sugar diet, could not be completely prevented by inulin or sodium butyrate, these dietary supplements have beneficial effects on gut health which can be partly attributed to their ability to ameliorate gut barrier function offering new approaches for the therapeutic use.

Prebiotic fibers such as inulin are metabolized by the human gut microbiota into the short-chain fatty acids (SCFA) butyrate, propionate and acetate (60, 61) and have been shown to promote SCFA production in obesity and type 2 diabetes (62, 63). The herein described favorable effects of inulin tended to be more pronounced than those of sodium butyrate suggesting that SCFAs other than butyrate are involved in inulin effects. Despite the generally accepted concept that inulin and inulin-type fructooligosaccharides (FOS) are not fermented in the small intestine, it is likely that inulin is partially fermented in the ileum. Several studies have shown that more than 10% of inulin and FOS are either hydrolyzed by acidic conditions or digested by microbiota in the distal small intestine (64–66). The ability to degrade inulin has been demonstrated for Bifidobacteria (67–70) as well as for Lactobacillaceae (71) which colonize the distal small intestine. We hypothesize that bacteria such as Bifidobacteria and Lactobacilli metabolize parts of the supplemented inulin and thus the arising SCFAs influence antimicrobial peptide expression in the ileum. Furthermore, this might be especially relevant in small intestinal bacterial overgrowth, which is promoted by diets high in saturated fats and sugar and is highly prevalent in NAFLD. This is corroborated by our findings that in mice fed a WSD or WSDF bacterial density was significantly higher compared to the CD fed mice.

Assessing the effects of sodium butyrate, propionate and acetate on expression of α-defensins in small intestinal crypts *in vitro* revealed that the bacterial metabolites induced α-defensin mRNA expression. Interestingly, MMP7 mRNA expression level were also augmented by sodium butyrate and propionate suggesting that the microbial metabolites are involved in the maintenance of intestinal homeostasis by inducing α-defensin secretion from Paneth cells. These findings align with a previously reported enhanced secretion of Defa1 in isolated mouse crypts in response to butyrate (42). We attained additional evidence that histone deacetylation and STAT3 might play a role in the transcriptional regulation and inducibility of α-defensins. Since we did not analyze the effects of broadband inhibition of HDACs (class I and IIa), but targeted class I HDAC1-3 and -8 inhibition, we cannot exclude that inhibition of further HDACs might play a role in the induction of Paneth cell α-defensin expression. However, it is very likely that SCFA regulate α-defensins *via* multiple mechanisms and future studies are necessary to address the mechanisms that underlie SCFA-induced up-regulation of α-defensins in more detail. Some of the protective effects of inulin are likely to be mediated not only by microbial metabolites after the bacterial fermentation of fiber but through changes in the microbiota composition.

Collectively our data indicate that inulin and sodium butyrate attenuate high-fat and high sugar-diet induced downregulation of α-defensins and contribute to the restoration of antimicrobial barrier function. Although future experiments are necessary to elucidate the mechanisms underlying the induction of α-defensin function in more detail, our results demonstrate promising efficacy of inulin and sodium butyrate as a dietary therapeutic strategy aiming to ameliorate gut barrier dysfunction and thereby reducing syndromes associated with diet-induced obesity.

## Data Availability Statement

The raw data supporting the conclusions of this article will be made available by the authors, without undue reservation.

## Ethics Statement

The animal study was reviewed and approved by Regional Council Stuttgart.

## Author Contributions

JB and SB designed the research. JB, LF, VK-V, and IS performed experiments. JB LF, and VK-V analyzed data. JB prepared figures and wrote the manuscript. IS and CG contributed materials and analysis tools and participated in scientific discussion. JB and LF edited and revised the manuscript. All authors approved final version of the manuscript and the submitted version.

## Funding

This work was funded in part by a grant from the German Research Foundation (DFG) BI 424/8-1 and Federal Ministry of Education and Research grant 01GI1122H (both to SB).

## Conflict of Interest

The authors declare that the research was conducted in the absence of any commercial or financial relationships that could be construed as a potential conflict of interest.
