# Supplementary material for: Prebiotic Inulin and Sodium Butyrate Attenuate Obesity-Induced Intestinal Barrier Dysfunction by Induction of Antimicrobial Peptides
Source: Front Immunol. 2021 Jun 11;12:678360. doi: 10.3389/fimmu.2021.678360 (PMC8226265; doi:10.3389/fimmu.2021.678360)
Supplement: Supplementary file 1 [file Table_1.docx]

Supplementary Material

# Supplementary Figures

**Supplementary Figure 1.** Energy intake and water intake were not affected by inulin or sodium butyrate supplementation to WSD. C57BL/6 mice fed either a control diet (CD), a Western-style diet (WSD) or a WSD supplemented with 10% inulin (inu) or 5% sodium butyrate (but) with or without additional fructose (F) are shown. Cumulated energy intake per mouse after the 12-week intervention period and average water intake per mouse per week are shown. Statistical analysis was performed by one-way ANOVA with Sidak´s post-test. Significant differences are indicated as * p-value < 0.05; ** p-value < 0.01.


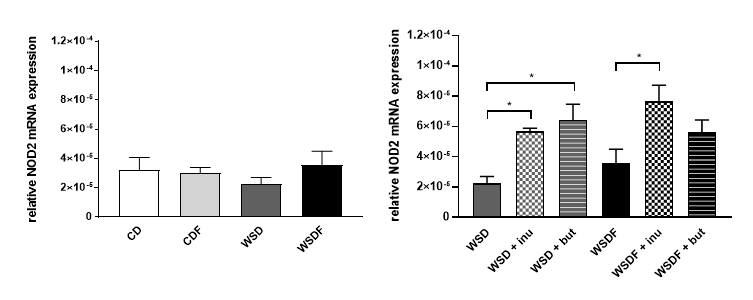
**Supplementary Figure 2.** Effect of inulin and sodium butyrate supplementation on mucin-2 expression. C57BL/6 mice fed different diets as described in Figure S1. Relative mRNA expression level of mucin-2 (Muc2) in the colon determined by quantitative RT-PCR. Data are presented as means +/- standard error of the mean (SEM) (n = 6–8). Statistical analysis was performed by one-way ANOVA with Dunnett´s post-test or by Kruskal-Wallis-test for non-parametric data with a Dunn’s post-test.

**
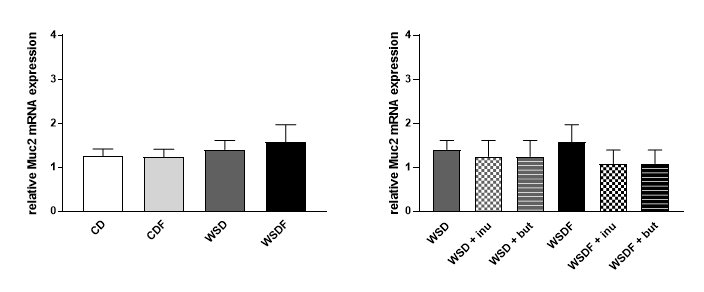
Supplementary Figure 3.** Effect of inulin and sodium butyrate supplementation on Nod2 expression. C57BL/6 mice fed different diets as described in Figure S1. Relative mRNA expression level of Nod2 in the ileum determined by quantitative RT-PCR. Data are presented as means +/- standard error of the mean (SEM) (n = 6–8). Statistical analysis was performed by Kruskal-Wallis-test for non-parametric data with a Dunn’s post-test. Significant differences are indicated as * p-value < 0.05.

**Supplementary Figure 4.** Effect of inulin and sodium butyrate supplementation on IL-1β expression. C57BL/6 mice fed different diets as described in Figure S1. Relative mRNA expression level of IL-1β in the ileum (A) and colon (B) determined by quantitative RT-PCR. Data are presented as means +/- standard error of the mean (SEM) (n = 6–8). Statistical analysis was performed by Kruskal-Wallis-test for non-parametric data with a Dunn’s post-test. Significant differences are indicated as * p-value < 0.05.

**Supplementary Figure 5.** Effect of inulin and sodium butyrate supplementation on GPR41, GPR43 and GPR109a expression. C57BL/6 mice fed different diets as described in Figure S1. Relative mRNA expression level of GPR41 in the ileum (A) and colon (C), GPR43 in the ileum (B) and colon (D) and GPR109a in the colon (E) determined by quantitative RT-PCR. Data are presented as means +/- standard error of the mean (SEM) (n = 6–8). Statistical analysis was performed by Kruskal-Wallis-test for non-parametric data with a Dunn’s post-test. Significant differences are indicated as * p-value < 0.05. ** p-value < 0.01
